# Conditioned medium from human adipose-derived mesenchymal stromal cells can modulate cell migration and morphology of keratinocytes in vitro

**DOI:** 10.1007/s13577-026-01353-9

**Published:** 2026-02-03

**Authors:** Cristiano Rodrigues, Thaís Casagrande Paim, Carla Zanatelli, Elisa Vasconcellos Soares Prignon, Jéssica Gonçalves Azevedo, Liliana Ivet Sous Naasani, Márcia Rosângela Wink

**Affiliations:** 1 Laboratório de Biologia Celular, Universidade Federal de Ciências da Saúde de Porto Alegre (UFCSPA), Rua Sarmento Leite, 245., Porto Alegre, RS CEP: 90050-170 Brasil; 2 Departamento de Ciências Básicas da Saúde, Universidade Federal de Ciências da Saúde de Porto Alegre (UFCSPA), Rua Sarmento Leite, 245., Porto Alegre, RS CEP: 90050-170 Brasil

**Keywords:** Human adipose-derived mesenchymal stromal cells, HaCaT, Cell migration, actin filaments, Nuclei irregularity, TGF-β, SB 431542

## Abstract

The regenerative ability of the skin is orchestrated by different types of cells, including mesenchymal stromal cells (MSCs). Their role in wound healing is being widely studied due to their capacity to produce a secretome able to modulate their microenvironment. In this context, MSCs factors can be isolated using in vitro cell culture and be experimentally tested for a series of clinical conditions, such as skin repair. In this study, we produced conditioned medium (MSC-CM) using primary cultures from human adipose-derived MSCs. This medium was used as treatment in a culture of keratinocytes cell line from adult human skin (HaCaT) to investigate the modulation of their dynamics. We analyzed cell proliferation, migration, and changes on cell morphology by labeling the actin filaments and nuclei. The factors released by MSCs were able to improve both the proliferation and migration of keratinocytes. In addition, there was an increase in the amount of actin stress fibers, filopodia protrusions, and nuclei irregularities. The MSCs secretome modified the migratory patterns of keratinocytes, being observable through their morphological changes. At least in part, this modulation was caused by the TGF-β1 signaling, considering that its antagonist, SB 431542, lead to a reduction of approximately 76% in the migration of HaCaT cells through the porous membranes of transwell chambers. Together, our data contribute to a better understanding of the role of MSCs on keratinocytes during wound healing and reinforce the importance to investigate their potential in dermal regeneration therapies.

**Supplementary Information:**

The online version contains supplementary material available at 10.1007/s13577-026-01353-9.

## Introduction

Wound healing is the process by which the body tissue repairs itself after a trauma. It involves a series of chemicals signaling, extracellular matrix deposition, proteolysis, and cellular proliferation. The re-epithelialization of wounded skin also requires the rapid and coordinated migration of keratinocytes into the wound bed to restore the mechanical barrier, allowing both the remodeling and the maturation of the regenerated tissue [1]. The program is modulated in a manner that is scaled to match the size of the wound, but this process can be delayed in cases of patients dealing with large lesions or critical clinical conditions [2]. Currently, new cell-based therapies using MSCs are being tested, and demonstrate potential in the field, by promoting the healing of burns or other mechanical traumas to the skin [3, 4].

MSCs naturally contribute to the process of healing. This subpopulation can be isolated from various sites of adult tissues, including the bone marrow, the adipose tissue, the umbilical cord, and the placenta [5]. In the skin, the MSCs that are residing around blood vessels, in the bulge area of hair follicles, and in the basal layer of epidermis may give rise to keratinocytes [6]. Interestingly, besides their multipotential, their capacity of producing paracrine factors has been suggested as the principal mechanism that contributes to tissue repair, since this production allows a modulation of their microenvironment [7, 8]. This secretome consists of an amount of proteins (growth factors and cytokines), microvesicles, or exosomes that can alter the cell’s homing, division, migration, differentiation, apoptosis and its immune status during wound healing [9]. Bioactive molecules such as the epidermal growth factor (EGF) and the transforming growth factor (TGF-β) are examples of compounds that may bind on keratinocyte receptors, hence triggering signaling pathways [10].

The TGF beta superfamily includes three isoforms (TGF-β1, 2, and 3), BMP (Bone Morphogenic Proteins), and activins [11]. Each TGF-β isoform may have a different role in wound healing, regulating cell proliferation and migration, differentiation, deposition and remodeling of the extracellular matrix, collagen synthesis and immune modulation [12]. The endogenous TGF and activin signaling pathway can be inhibited in vivo by SB-431542, a specific inhibitor of activin receptor-like kinase (ALK) receptors ALK4, ALK5, and ALK7. SB-431542 inhibits the capacity of activated ALK4, ALK5, and ALK7 to promote both Smad2/Smad4- and Smad3/Smad4-dependent transcription [13].

Through in vitro cell culture, the trophic factors secreted by MSCs can be accessed and tested on target cells. The influence of MSCs on keratinocytes is currently being analyzed to understand better the role of MSCs during skin repair. This knowledge aims to enable their safe and satisfactory employment in regeneration therapies [14]. Therefore, in this study, we have examined the healing capacity of the conditioned medium from adipose-derived MSCs on an in vitro skin wound healing model, using a confluent monolayer of HaCaT keratinocytes, complemented by a co-culture in a transwell system. Through these analyses, we have reiterated that the MSCs secretome can modulate the keratinocyte migration, at least in part, through the TGF-β signaling.

## Materials and methods

(For detailed information of material and methods, please see the expanded material and methods file).

### Hacat cell line

The immortal keratinocytes from adult human skin (HaCaT) acquired from the ATCC (Manassas, Virginia, USA) collection were kindly provided by Dr. Jenifer Saffi (UFCSPA, Brazil).

### Human adipose-derived MSCS primary culture

#### Cell isolation and Adipose-derived MSCs differentiation

Human adipose-derived MSCs were isolated from the abdominal adipose tissue of 3 healthy female donors (aged 31–45 years), then cultured and characterized as described by Naasani et al. (2019) [15].

### Preparation of MSC-CM

The conditioned medium was performed by seeding MSCs between the passages 4 to 9, at a density of 3320 cells/cm^2^, on plastic culture flasks, to form a semi-confluence [16]. After 24 h of culture with DMEM 10% FBS, the cells were washed once with PBS and covered with 120 µL/cm^2^ of serum-free DMEM medium to release factors for the next 24 h under an appropriate atmosphere (humidified, 5% CO_2,_ 37°ºC). The resulting MSC-CM was collected and then filtered, through centrifugation (600 × *g*, 10 min) to eliminate the cellular debris. It was then stored at a temperature of − 80 °C until further use. The MSC-CM obtained from each patient was considered an independent sample for subsequent analyses.

### Proliferation assay

Cell counting was performed by 0.4% trypan blue exclusion method. 1 × 10^5^ HaCaT cells was seeded in a 24-well plate, remaining for 24 h for cell adhesion. The monolayers were washed and exposed to the treatments: DMEM 10% FBS, MSC-CM, and serum-free DMEM remaining for 24, 48, 72 and 96 h. Cultures pretreated with 20 µg/mL mitomycin C (Sigma) for 4 h were also used as a complementary control for the two initial times. The last two groups had their medium changed on the second day, when the nonadherent cells were collected and counted. All cultures tested were washed with PBS, detached using 0.25% trypsin and 0.01% EDTA in PBS (*v/v*), and dissociated for counting. The numbers of viable and dead cells were assessed by counting with a Neubauer chamber under a phase-contrast microscope (DMi1, Leica).

### Scratch wound assay and transwell migration assay

To proceed with the migration assay, HaCaT cells were plated on 24-well plates to obtain a final density of 1 × 10^5^ cells per well, as described by Liang (2007) [17]. To ensure that the observed gap closure was due only to migration and not cell proliferation, the HaCaT cells were pre-treated for 2 h with 20 µg/mL Mitomycin C (Sigma-Aldrich) prior to injury. Complementary, the migration of HaCaT cells was also evaluated during a co-culture with MSCs using transwell inserts (Greiner Bio-One), as described by Iser (2016) [16]. Specifically, HaCaT cells were plated in the upper chamber of transwell inserts featuring an 8 µm pore size membrane, while the MSCs were plated in the corresponding lower well, acting as the chemoattractant source. For further technical specifications, please refer to the Expanded Materials and Methods document.

### Actin cytoskeleton staining

The actin cytoskeleton reorganization was assessed via filamentous actin (F-actin) staining. Briefly, HaCaT cells were seeded on 13 mm glass coverslips to obtain a final density of 4 × 10^4^ cells per well, then were maintained for 24 h with the DMEM 10% FBS to allow cell adhesion. After this, the cells were treated for periods of 24 and 48 h, either with the MSC-CM or one of the controls: DMEM, DMEM 10% FBS, DMEM 10 ng/mL TGF-β1, and MSC-CM 2 µM SB 431542.

### Nuclear morphometric analysis (NMA)

The nuclear morphology of the HaCaT cells was analyzed using the NMA software developed by Filippi-Chiela and co-authors [18]. Briefly, after having undergone the processes of seeding and adhesion, cells were either treated with the DMEM 10% FBS, the serum free culture medium, or the MSC-CM for periods of 24 and 48 h, fixed and analyzed as already described [4].

### TGF-β1 quantification by enzyme-linked immunosorbent assay (ELISA)

The concentration of Transforming Growth Factor-beta 1 (TGF-β1) in the MSC-conditioned medium (MSC-CM) was determined using the Human TGF Beta 1 (PicoKine® ELISA Kit, Boster Biological Technology). The assay was performed according to the manufacturer's protocol, which quantifies total TGF-β1 (active and latent forms). Briefly, MSC-CM samples were subjected to an acid activation step to release the active form from the Latency-Associated Peptide (LAP). Activated samples, along with control medium and a recombinant human TGF-β1 standard curve, were added in duplicate to the pre-coated microplate wells. Following the appropriate incubation and washing cycles, a biotin-labeled detection antibody and Streptavidin-HRP conjugate were sequentially added. The reaction was developed using TMB substrate, and stopped by the addition of stop solution. The absorbance was read at 450 nm using a microplate reader (SpectraMax, Molecular Devices). The concentration of TGF-β1 was calculated by interpolating the sample optical density values from the standard curve.

### Statistical analysis

The data were analyzed through the one-way or two-way ANOVA followed by Bonferronis’s or Tukey’s multiple comparisons test, using the Graph Pad Prism 6 software (La Jolla, CA, USA). The results are expressed as mean value ± standard deviation. The differences were considered significant when the *p* value was of 0.05 or less.

## Results

### Mesenchymal stromal cell characterization

The collection of the adipose tissue, for the cell isolation, is less traumatic than other tissues, and the presence of MSCs is higher in fat [19]. Thus, we chose the adipose tissue as a source of these cells. To improve the uniformity of the assays, the donors screened normal body mass indexes. The MSCs were isolated from the abdominal adipose tissue of three independent female healthy donors.

**Fig. 1:**
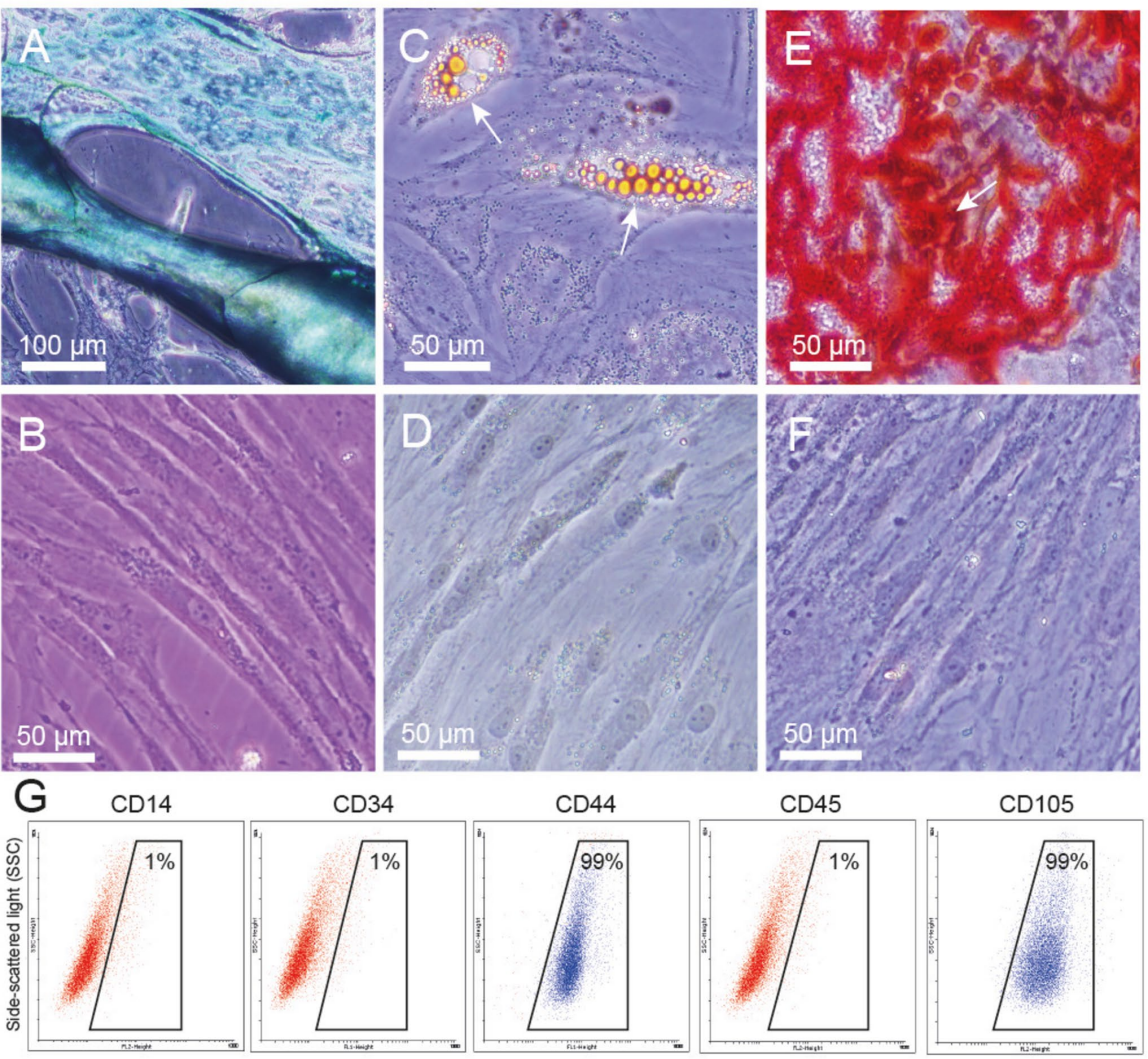
Characterization of adipose-derived MSCs. MSCs underwent differentiation in **A** chondrocytes stained by Alcian blue; **C** adipocytes, showing with white arrows some lipid vesicles stained by Oil Red O; and **E** osteoblasts, showing calcium phosphate deposited on the culture, stained with Alizarin Red S, after 4 weeks of induction. Primary cultures of MSCs had fibroblast–like shapes and demonstrated plastic adherent properties. Negative controls of differentiation in the absence of induction medium **B**, **D** and **F** were also dyed according to their peers. The absence of stain although each being exposed to a different dye showed the MSCs presented stemness maintenance under standard conditions at the same time as the inducted cultures. **G** Dot plots of flow cytometry, showing that these undifferentiated MSCs were homogeneously positive for mesenchymal markers: CD44, CD105, and CD90; but negative for hematopoietic markers: CD14, CD34, and CD45

The adipose-derived MSCs exhibited an elongated fusiform morphology (fibroblast-like), self-renewal potential and plastic adherence, as observed in the controls of the differentiations (Fig. 1). Their multipotency was demonstrated by incubating the cells in media capable of promoting differentiation into the chondrogenic, adipogenic, and osteogenic lineages (Fig. 1A, C and E, in comparison with controls 1B, D and F). Blue staining indicates the synthesis of proteoglycans by chondrocytes (Fig. 1A). The MSCs also differentiated into adipocytes or osteoblasts, as evidenced either by the presence of intracellular lipid droplets (Fig. 1C), or by calcium-rich mineralized matrix deposits (Fig. 1E). The analysis of the phenotype, by flow cytometry, showed that these cells expressed CD44, CD105, and CD90 proteins; and were negative for the hematopoietic markers CD14, CD34, and CD45 (Fig. 1G). The immunophenotype profile and differentiation potential obtained are consistent with recent guidelines for the characterization of MSCs. After confirming the identity of the MSCs, we proceeded with the preparation of the MSC-CM to be tested in the next experiments.

### MSC-CM improve keratinocyte proliferation

**Fig. 2:**
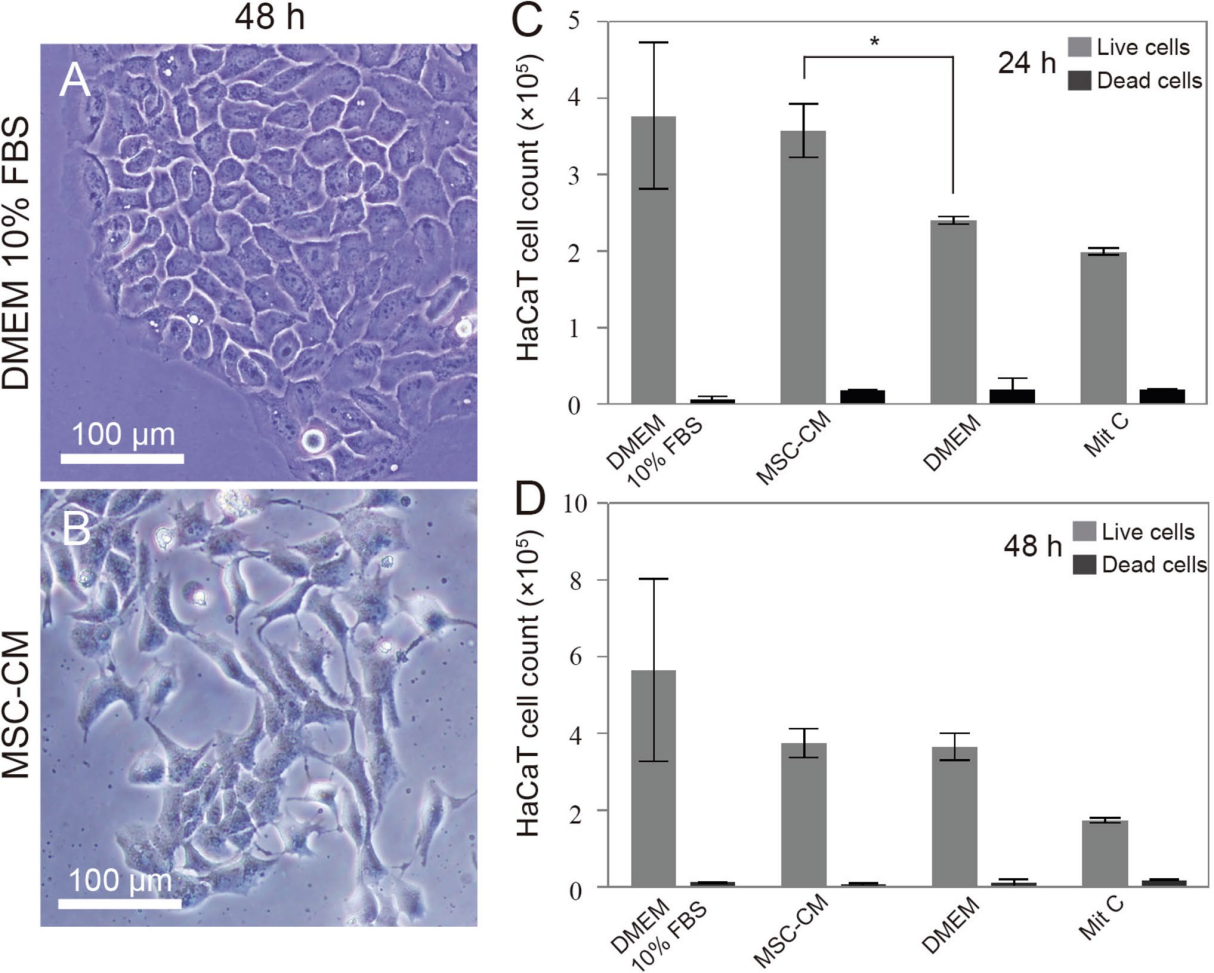
HaCaT cell morphology and proliferation patterns. **A** When cultured in standard medium, epithelial-like cells are polygonal in shape, showing more regular dimensions, and grow attached such as defined colonies. But, after 48 h of contact with MSC-CM, **B** HaCaT cells alter their morphology, turning more elongated and losing the juxtaposition of the colony. **C** The proliferation rate of HaCaT cells was increased after 24 h *(*p* = 0,0171) of MSC-CM exposition, but not in 48, 72, and 96 h, as shown in the cell count charts. Data represents mean ± SEM of 3 independent experiments. Mit C: mitomycin C was used as a control to stop cell division. DMEM 10% FBS: standard medium. DMEM: serum-free medium. MSC-CM: adipose-derived MSCs conditioned medium

When observed by optical microscopy, the cell morphology of HaCaT cultures treated with MSC-CM for 48 h revealed a change from the characteristic pattern, being more elongated, with non-juxtaposed cells, when compared with cells that grew in the standard culture (Fig. 2A, B). In addition, keratinocytes treated with MSC-CM exhibited increased proliferation rates compared to the negative control (DMEM) at 24 h, but not at 48, 72, or 96 h (Fig. 2C). There was an increase of 156.1 ± 71.9% in the number of cells, when HaCaT culture received the conditioned medium for 24 h, while serum-free medium, used as control, showed a rise of 70.9 ± 35.6%.

### MSC-CM enhances keratinocyte migration

**Fig. 3:**
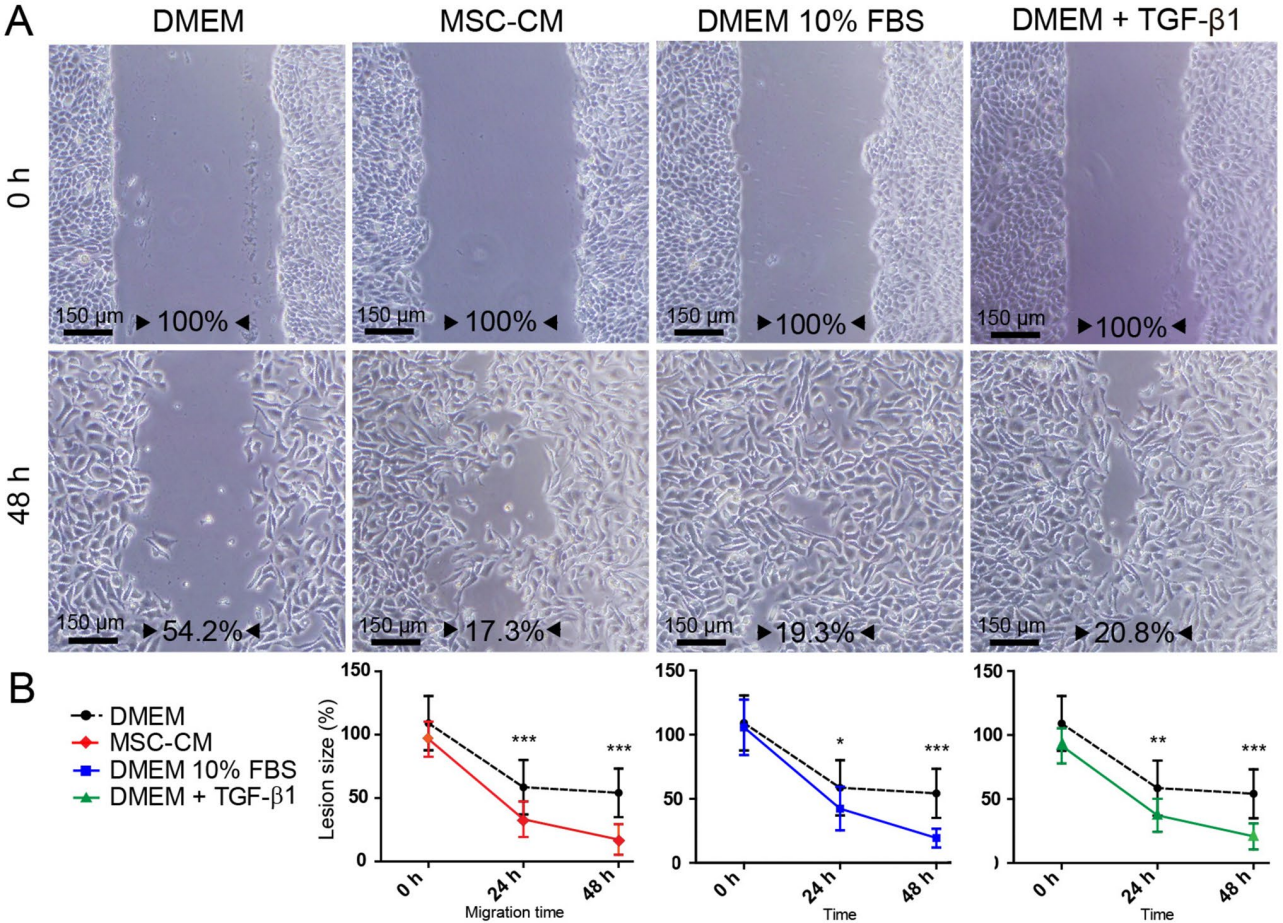
Migration of HaCaT cells by in vitro scratch wound assay. **A** photomicrographs by optical phase-contrast microscopy (40 × magnification) of lesions scratched in a keratinocyte monolayer under different treatments followed for 48 h after injury. The wound width had an average of 480.5 µm, reaching Ø15.4 mm of cell culture. Percentages refer to the lesion area still open, according to time and treatment. **B** Graphs show differences in the lesions size that received the treatments: MSC-CM, DMEM 10% FBS and 10 ng/mL TGF-β1 in DMEM (solid lines) at 24 and 48 h, in comparison with the negative control: serum-free DMEM (dashed lines). Scale bar: 150 µm. DMEM: serum-free medium. DMEM 10% FBS: standard medium. MSC-CM: adipose-derived MSCs conditioned medium. Data represents mean ± SEM of n: 3 independent experiments. Significance compared to the negative control at 48 h: MSC-CM (*p* = 0,00045), DMEM 10% FBS (*p* = 0,00074), and TGF-β1 (*p* = 0,0009). TGF-β1: transforming growth factor beta I

HaCaT cells treated with the MSC-CM migrated and closed the wound more rapidly, in the scratch wound assay, than those that received the serum free control medium (DMEM) (Fig. 3A and B). Considering the time zero (0 h) as 100% of scratch opening, at 24 h, the extent of the wound was 33.2 ± 14% in presence of the MSC-CM and 58.5 ± 21.4% in the DMEM. After 48 h, the MSC-CM treatment showed 17.3 ± 12% of open lesion, compared to 54.2 ± 19.1% for the control medium. Furthermore, we tested as positive control the standard culture medium of HaCaT cell (DMEM 10% FBS) that showed an overage of 42.3 ± 16.9% in 24 h, and 19.3 ± 7.2% in 48 h. In order to investigate whether the TGF-β1 signaling was involved in the modulation of the migration, we treated HaCaT cells with 10 ng/mL of TGFβ−1 factor. Indeed, the results showed that TGFβ−1 was able to enhance the HaCaT migration, similarly to the levels of the DMEM 10% FBS (Fig. 3B).

**Fig. 4:**
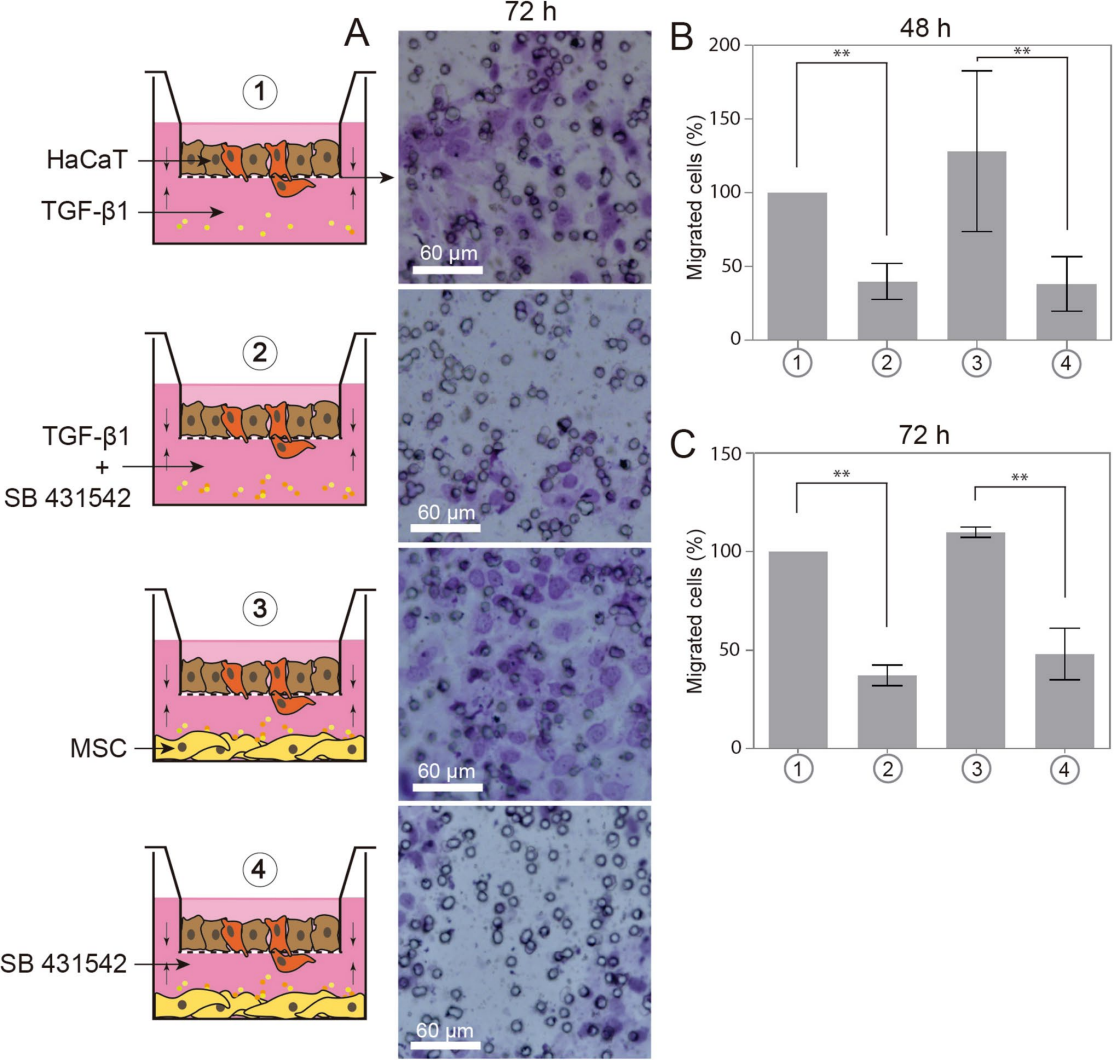
Filopodia protrusions in keratinocytes. Fluorescence microscopy analysis at 24 and 48 h after the MSC-CM treatment. A sheet of keratinocytes, in a scratch wound assay, exhibits the extensions of filopodia (arrows) moving the leading edge of the cells toward the empty space of the wound site. Graphs show the number of filopodia per cell at 24 and 48 h of migration on the scratched area. There is an increase of filopodia protrusions at 48 h *(*p* = 0.003), when comparing the MSC-CM with the control medium (FBS deprivation). Data represents mean ± SEM of 3 independent experiments. DMEM: serum-free medium. MSC-CM: adipose-derived MSCs conditioned medium. DMEM 10% FBS: standard medium

During the scratch wound closure, keratinocytes treated with MSC-CM exhibited an increase in filopodia protrusions directed toward the wound at 48 h, compared to the negative control (Fig. 4).

### MSC-CM modifies nuclear morphology

**Fig. 5:**
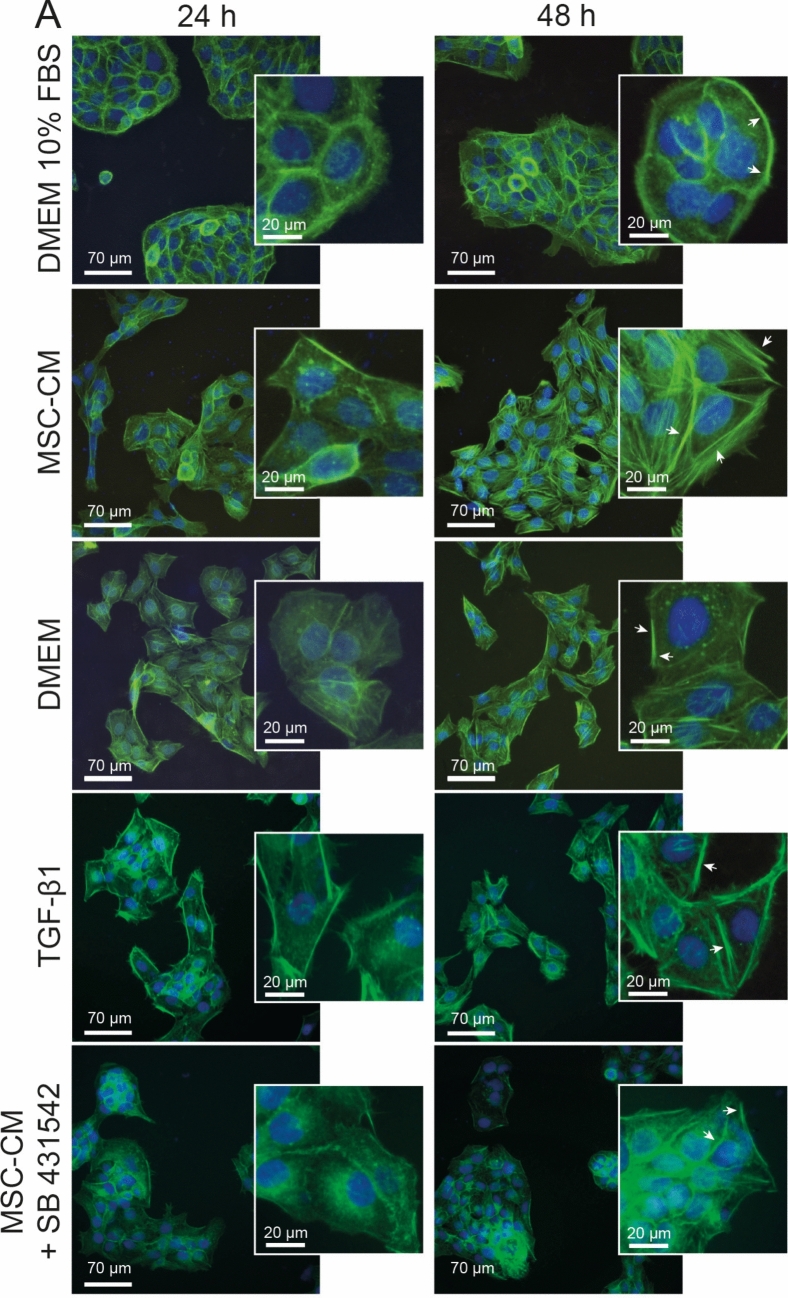
Morphological nuclear changes in HaCaT cells. **A** Fluorescence microscopy with DAPI staining followed by the scatter plot of area (µm^2^) versus nuclear irregularity index (NII), after 24 h of treatment with DMEM, MSC-CM, and DMEM 10% FBS. **B** Fluorescence microscopy and plot of NII after 48 h of treatment. **C**, **D** Graphics show the number of irregular nuclei after 24 and 48 h. Keratinocytes exhibited an increase of irregular nuclei after 48 h of treatment with the MSC-CM ***(*p* = 0,0002), when compared with the standard culture medium. Data represents mean ± SEM of 3 independent experiments. Normal nuclei (NN), irregular nuclei (IN). DMEM 10% FBS: standard medium. MSC-CM: adipose-derived MSCs conditioned medium. DMEM: serum-free medium

We analyzed the cell nucleus morphology as a complementary parameter to the keratinocyte behavior change (Fig. 5A and B). The MSC-CM treatment could increase the number of irregular nuclei from 3 ± 0.5% at 24 h, to 16 ± 1% at 48 h, on the studied cell population. The standard culture medium changed from 1 ± 0.3% at 24 h, to 6 ± 0.7% at 48 h (Fig. 5C and D). Also, cells that received the DMEM without FBS showed 0.9 ± 0.3% of irregular nuclei at 24 h, and 7 ± 1% at 48 h of culture. In the end, the cell population treated with the MSC-CM for 48 h presented a significant difference compared to the cells that received the standard medium (DMEM 10% FBS) at the same time (Fig. 5B and D).

### MSC-CM alter actin cytoskeletal organization

**Fig. 6:**
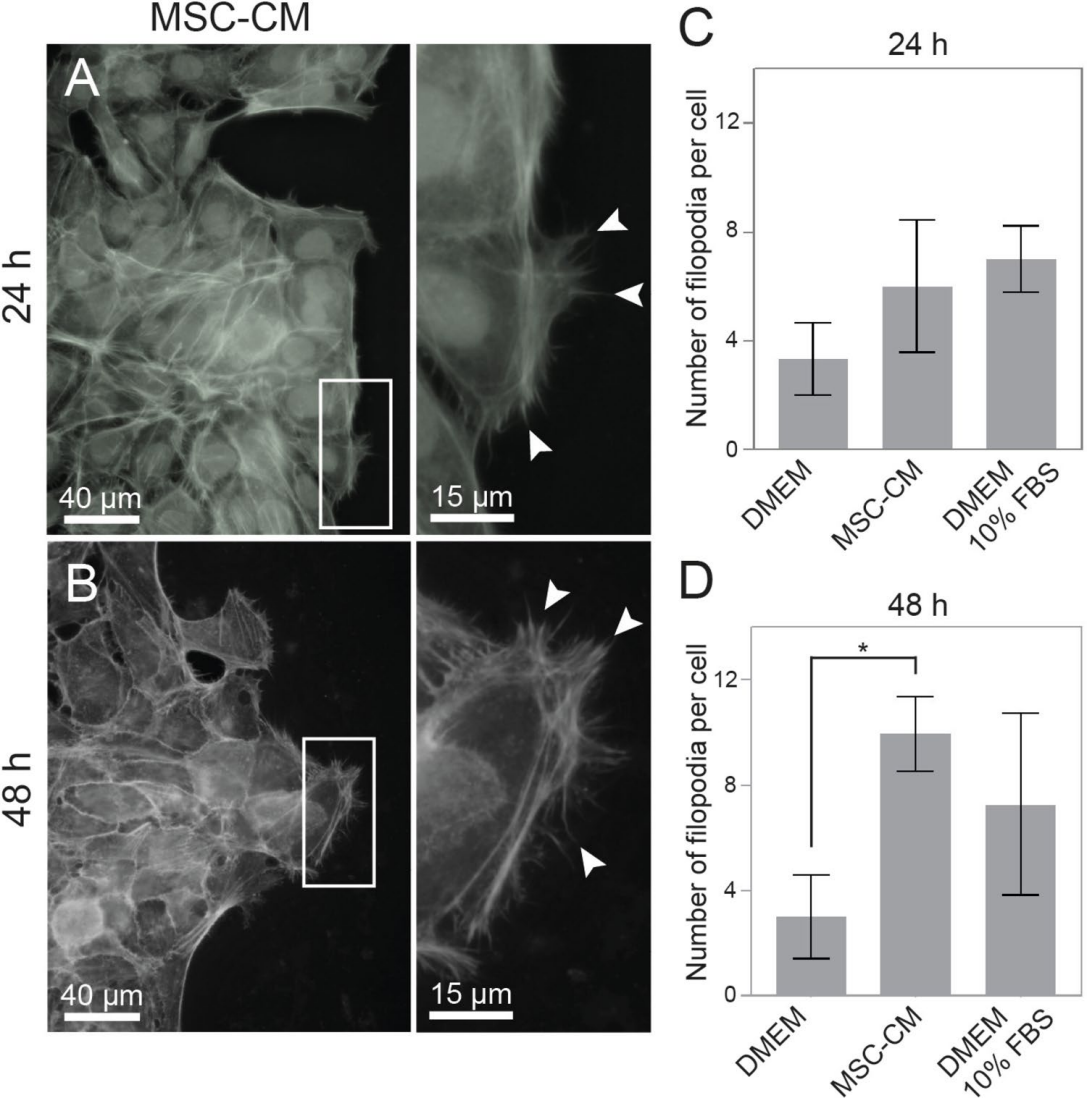
Actin cytoskeleton of keratinocytes. **A** Representative images of colonies after different treatments at 24 and 48 h. Keratinocytes that received the standard culture medium, exhibited a typical morphology, showing actin fibers concentrated at the cell periphery with maintenance of cell–cell contacts, and yet extending their lamellipodia. However, all those with other treatments showed some morphological alterations. Both MSC-CM and TGF-β1 treatments modulated the actin cytoskeleton, showing higher amount of stress fibers in the cell body. Cultures that received FBS deprivation presented a decrease in the visible fibers of stress and in the concentration in cellular cortexes, following a reduction of both protrusive structures: lamellipodia and filopodia. DMEM 10% FBS: standard medium. MSC-CM: adipose-derived MSCs conditioned medium. DMEM: serum-free medium. TGF-β1: transforming growth factor beta type I. SB 431542: TGF-β1 receptor kinase inhibitor

In order to confirm the participation of TGF-β1in the morphological changes of HaCaT cells, the Phalloidin was incorporated into the F-actin filaments to observe the cytoskeletal network in the presence of SB 431542 (Fig. 6). Cells that received the standard medium (DMEM 10% FBS) exhibited circumferential and marginal bundles, forming dense transverse arcs after 24 and 48 h of culture, revealing normal cell characteristics patterns. The MSC-CM treatment, similarly to the treatment with TGF-β1 lead to depolymerization of actin filaments, demonstrating an increase of dorsal and ventral stress fibers, mostly at 48 h. The control HaCaT cells treated with serum free medium and MSC-CM plus SB 431542 showed some similarities, such as the reduction in transversal arcs, few ventral stress fibers and a low value of filopodia protrusions. However, both treatments presented morphological differences when compared to the standard culture of these cells.

### Transwell assay

**Fig. 7:**
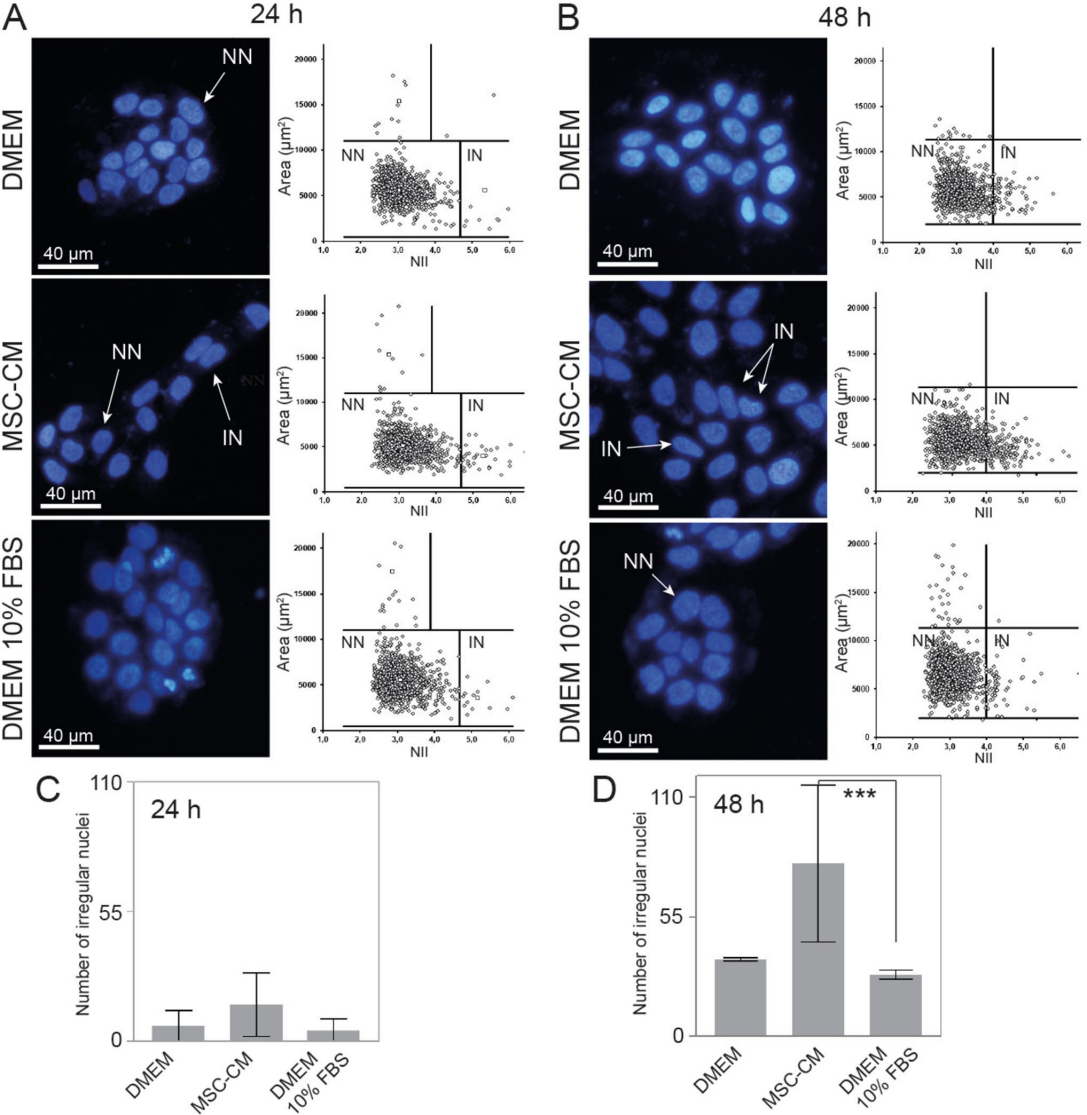
Migration of HaCaT cells by Transwell assay. **A** Scheme showing the different configurations of chemoattractant in the lower chambers. Aside, photomicrographs (200 × magnification) by optical microscopy of insert membranes, showing the density of migrated cells labeled by crystal violet staining, after 72 h of culture. Representative graphic of percent of migrated cells in **B** 48 and **C** 72 h, assuming TGF-β1 factor as a control of 100% of migration. The culture of MSCs under serum-free conditions could increase **(*p* = 0.0035) the number of HaCaT migrating cells at both times (74 ± 69 in 48 h and 110 ± 2.6 migrated cells in 72 h), as much as the control TGF-β1 (84.4 ± 83 in 48 h and 89 ± 88.6 in 72 h). By another way, the effect was blocked by the concomitant treatment with the SB 431542 inhibitor. Data represents mean ± SEM of 3 independent experiments. DMEM 10% FBS: standard medium. MSC-CM: adipose-derived MSCs conditioned medium. TGF-β1: transforming growth factor beta I. SB 431542: TGF-β1 receptor kinase inhibitor

Thus, to confirm whether TGFβ−1 was present in the MSC-CM and that it was one of the factors involved in the increase of migration observed in the scratch wound assay, we proceeded with a more sophisticated assay, using the transwell method. In this system, HaCaT cells were grown on top of transwell chambers and MSCs in the bottom of plate (scheme of the Fig. 7A). Under culture on serum-free conditions, in 24 h as well as in 48 h, the MSCs were able to promote the migration of HaCaTs at the same level than the TGF-β1 factor, used to set the positive control (100% value) (Fig. 7B and C). The migration in these two conditions was partially blocked by SB 431542. The presence of SB 431542 inhibited the HaCaT migration by 75 ± 28.5% and 77.4 ± 19.4%, in 48 h and in 72 h, respectively.

### Quantification of TGF-β1 in the MSC-CM

The ELISA analysis demonstrated that the MSC-CM contained a total TGF-β1 of 8.6 ± 3 pg/mL (Supplementary Fig. 1). Although the quantification yielded a low concentration, it is consistent with the established high biological potency of TGF-β1, which is active in the picogram range.

## Discussion

In this study, we evaluated the influence of MSC-CM on the mechanical response of HaCaT keratinocytes, focusing on key parameters such as morphology and migration. These aspects are crucial for biological processes like embryogenesis, tissue morphogenesis, and wound healing [20]. Given the growing interest in cell therapies for cutaneous wound repair, HaCaT cells serve as an important model due to their distinct genetic characteristics [21]. They are capable of differentiating and forming an epidermal equivalent in vitro using three-dimensional methods [22] and, when transplanted into nude mice, do not exhibit tumorigenic potential, instead responding to skin wound control mechanisms [23, 24]. These characteristics make HaCaT cells widely accepted in preliminary dermatologic studies.

More than 30 years ago, Friedenstein and collaborators first identified fibroblast-like cells that could be isolated from bone marrow via adherence to plastic surfaces [25]. Today, it is well established that MSCs reside in virtually all postnatal human tissues, contributing to tissue homeostasis (Klimczak & Kozlowska 2016). Their ability to differentiate into specialized cells and produce key molecules for healing makes them highly relevant for cell-based therapies (Deng et al. 2024). MSC-conditioned medium (MSC-CM) has been widely studied to assess its effects on other specialized cells [26, 27] and can be generated under various conditions, including three-dimensional cultures, hypoxic environments, or differing fetal bovine serum (FBS) concentrations [28, 29]. In this study, MSC-CM was prepared under standard atmospheric conditions (humidified, 37ºC, 5% CO₂), adhered to plastic surfaces at semi-confluence, and under FBS deprivation, considering that serum-derived factors might overlap with MSC-secreted molecules.

Upon skin injury, MSCs are activated and exhibit tropism toward the lesion site, playing a crucial role in wound healing. Keratinocytes, in response, may lose their epithelial characteristics and acquire a mesenchymal phenotype, becoming less adhesive and more migratory, thereby accelerating barrier restoration. TGF-β1 has been identified as a key regulator of this transition, promoting morphological changes in epithelial cells [30].

In our experiments, MSC-CM significantly increased keratinocyte migration and induced morphological alterations, even in short periods. The wound created via scratch assay in a HaCaT monolayer closed significantly faster under MSC-CM treatment. When HaCaT cells were co-cultured with MSCs in serum-free conditions, they exhibited directed migration toward MSCs, similar to responses observed when using TGF-β1 as a chemoattractant. This migratory effect was partially blocked by treatment with SB-431542, confirming that TGF-β1 is one of the soluble factors secreted by MSCs and plays an essential role in cell migration [30].

The profound dependence of the migratory effect on the TGF-β signaling pathway, as evidenced by the significant ~ 76% reduction in migration following SB-431542 treatment, suggests that the underlying mechanism extends beyond a straightforward paracrine stimulus from the MSC-CM. It is plausible that the initial TGF-β1 signal supplied by the secretome induces the HaCaT keratinocytes to initiate an autocrine amplification loop. Activated keratinocytes are well-documented to produce and secrete their own TGF-β isoforms, which subsequently bind to cell-surface receptors to self-sustain the migratory and phenotypic activation [31, 32]. This autocrine circuit is crucial for efficient re-epithelialization and offers an explanation for the robust inhibitory effect observed with the SB-431542 antagonist, as it effectively targets both the exogenous MSC-CM signal and the endogenous, amplified autocrine component.

Supporting our findings, Xuan et al. (2017) demonstrated that SB-431542 can block TGF-β1 expression and function, inhibiting human umbilical cord MSC (hUC-MSC) proliferation and contributing to hepatic anti-fibrosis via the TGF-β1/Smad pathway [33]. Additionally, SB-431542 was reported to inhibit B16 melanoma cell migration and invasion, reinforcing its role in suppressing EMT induction [34]. Other studies also highlight the migratory effects of MSC-secreted factors on keratinocytes. Walter et al. (2010) observed significant wound closure in scratched HaCaT monolayers following 27 h of MSC-CM treatment, attributing this effect to MSC-derived TGF-β1, IL-6, and IL-8 [27]. Ruiz-Cañada et al. (2017) further demonstrated that HaCaT cells, either wild-type or knockout for TGF-β receptor, exhibited differential migration responses, suggesting an optimal threshold for TGF-β signaling activation [35].

TGF-β not only regulates gene expression but also modifies cytoskeletal dynamics, promoting both loss of epithelial markers and activation of mesenchymal traits [36]. EMT-induced phenotypic changes enable epithelial cells to detach from organized tissue structures and migrate through the extracellular matrix [37]. F-actin polymerization is crucial for cell protrusion, generating tensile forces that facilitate cell contractility [38].

Here, we demonstrated that MSC-CM modulates actin fiber formation and movement in HaCaT keratinocytes. Under standard conditions (DMEM 10% FBS), keratinocytes exhibit peripheral actin stress fibers, supporting cell–cell junctions and reinforcing their epithelial identity. Following MSC-CM treatment, we observed a redistribution of actin fibers, with a decrease in peripheral support structures and an increase in centrally localized stress fibers, correlating with enhanced migratory capacity. These effects were replicated upon TGF-β1 stimulation, but not when its receptors were blocked, further confirming TGF-β1’s role in cytoskeletal reorganization.

Despite the promising findings demonstrated in vitro, we acknowledge that the present study possesses limitations inherent to the two-dimensional (2D) model utilized. The in vivo wound environment is characterized by a complex microenvironment, including a three-dimensional (3D) extracellular matrix, oxygen gradients, and the presence of various immune cells. In particular, hypoxic conditions and 3D culture systems are known to drastically modulate the secretome of MSCs, enhancing the release of factors such as VEGF, FGF, and immunomodulatory cytokines, which may be more relevant for tissue regeneration. The preparation of our MSC-CM under standard 2D, normoxic conditions, while serving as an initial validation standard, may underestimate the full therapeutic potential of MSCs [39, 40]. Therefore, our results should be interpreted within the context of a simplified in vitro system. Future studies focusing on secretome optimization in 3D bioreactors or under hypoxic conditions, followed by validation in preclinical in vivo models, are necessary steps to fully determine the clinical efficacy of MSC-CM in wound healing.

In summary, this report provides evidence that TGF-β1 secreted by human adipose-derived MSCs is a key factor in regulating the morphology and promoting the spreading of human keratinocytes. Therefore, recognizing the critical role of the MSC secretome in wound healing is essential for establishing the safety and effectiveness of cell-based therapies. These findings reinforce the potential of utilizing MSC-CM as a promising cell-free therapeutic alternative, which could be clinically applied as a safe and effective adjunctive treatment for tissue regeneration in chronic wounds.

## Supplementary Information

Below is the link to the electronic supplementary material.

Supplementary file1 (TIF 31 KB)

Supplementary file2 (DOCX 12 KB)

## Data Availability

The data supporting the findings of the current study can be obtained by contacting the first author.
