# Supplementary figures and images for: Conditioned medium from human adipose-derived mesenchymal stromal cells can modulate cell migration and morphology of keratinocytes in vitro
Source: Hum Cell. 2026 Feb 3;39(2):42. doi: 10.1007/s13577-026-01353-9 (PMC12864314; doi:10.1007/s13577-026-01353-9)

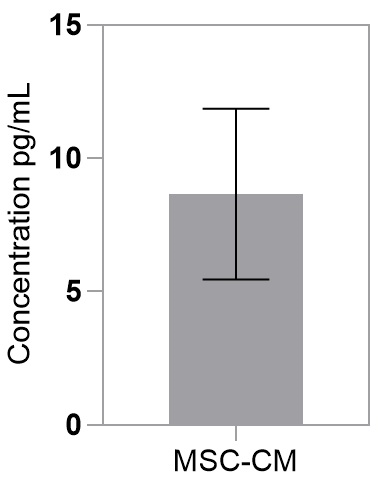

Supplement: Supplementary file 1 — Supplementary file1 (TIF 31 KB) [file 13577_2026_1353_MOESM1_ESM.tif]
