# Supplementary material for: Conditioned medium from human adipose-derived mesenchymal stromal cells can modulate cell migration and morphology of keratinocytes in vitro
Source: Hum Cell. 2026 Feb 3;39(2):42. doi: 10.1007/s13577-026-01353-9 (PMC12864314; doi:10.1007/s13577-026-01353-9)
Supplement: Supplementary file 2 — Supplementary file2 (DOCX 12 KB) [file 13577_2026_1353_MOESM2_ESM.docx]

**EXPANDED MATERIALS AND METHODS**

**HACAT CELL LINE**

The immortal keratinocytes from adult human skin (HaCaT) acquired from the ATCC (Manassas, Virginia, USA) collection were kindly provided by Dr. Jenifer Saffi (UFCSPA, Brazil). The cells were grown in culture flasks and maintained in a complete culture medium (DMEM 10% FBS) composed of Dulbecco’s modified Eagle’s medium supplemented with 4500 mg/L glucose, 10 mM HEPES, 23.8 mM NaHCO_3_, 100.000 U/mL penicillin, 100 mg/mL streptomycin (Sigma, St. Louis, MO, USA), and 10% *(v/v)* fetal bovine serum (FBS) (Cultilab, São Paulo, SP, Brazil). The cultures were kept at a temperature of 37 °C in a humidified 5% CO_2_ atmosphere and were routinely passaged at preconfluency using 0.25% trypsin and 0.01% EDTA (Invitrogen, Waltham, Massachusetts, USA).

**HUMAN ADIPOSE-DERIVED MSCS PRIMARY CULTURE**

*Cell isolation*

Human adipose-derived MSCs were extracted from the abdominal adipose tissue of female healthy donors (ages between 31-45 years) with normal body mass indexes (BMI). The patient consent was obtained before the liposuction and all protocols were approved by the Research Ethics Committee (REC-ISCMPA No.882968) from the Santa Casa de Misericórdia de Porto Alegre hospital (Porto Alegre, RS, Brazil), according to ethical principles expressed in the Declaration of Helsinki. The MSCs were isolated as already described (Naasani et al., 2019). Briefly, the fat fragments were washed and digested with collagenase solution (Type I, 1 mg/mL solubilized in DMEM), and were incubated in a water bath (37ºC) for 30 min. The cells were then centrifuged at 600 × *g* for 10 min. Next, they were resuspended in an erythrocyte lysis solution (150 mM NH_4_Cl, 10 mM NaHCO_3_ and 1 mM EDTA) diluted in ultrapure H_2_O 1:1 *(v:v)*. After a new centrifugation (600 × *g*, 10 min), the cells were resuspended in a known volume of DMEM 10% FBS and a quantity of 5.2 x 10^3^ cells/cm^2^ was then seeded in six-well plates and was cultured at 37ºC in a humidified incubator filled with 5% CO_2_, while covered by 3 mL of DMEM 10% FBS (1000 mg/L glucose). The first change of the medium took place 48 h later, when adherent cells appeared, and it was repeated each 4 days [(Sous Naasani et al. 2019)](https://paperpile.com/c/AA6mHs/UcD3).

*Adipose-derived MSCs differentiation*

The MSCs differentiation was performed, between the passages 4 to 7, at 2.2 × 10^5^ cells/cm^2^, in a specific medium (Gibco, Grand Island, NY, USA) meant for chondrogenic, adipogenic and osteogenic inductions. The cells were then cultivated for 4 weeks and the medium was changed every 3 days. After this period, the cells were washed once with PBS and were fixed with 4% *(v/v)* paraformaldehyde. For the chondrogenic assay, the cultures were stained with a 1% Alcian Blue solution. The adipogenic differentiation was processed with a 3.4% Oil Red O solution. The osteogenic was completed with 2% Alizarin Red S. The stained induced cultures, including the controls, were compared by assessing the dye impregnation. The photomicrographs were performed using a BX-50 Olympus microscope with optical lens (20X/0.30 Ph1-UplanFI) coupled with a camera Motican 2500 (Olympus, Tokyo, Japan) [(Rodrigues et al. 2014)](https://paperpile.com/c/AA6mHs/NlNe).

*Adipose-derived MSCs identification markers*

In addition, the surface markers of the MSCs were analyzed. The cells were dissociated with trypsin, centrifuged, and then incubated for 30 min at 4ºC using either phycoerythrin (PE) or fluorescein isothiocyanate (FITC), conjugated antibodies specific to the human CD14, CD34, CD44, CD45, CD105, and CD90 proteins (Invitrogen, Waltham, MA, USA). Analyses were performed using a FACSCalibur flow cytometer equipped with a 488-nm argon laser (Becton-Dickinson, San Diego, CA, USA) and with the CellQuest software. At least 10 000 events were collected [(Sous Naasani et al. 2019)](https://paperpile.com/c/AA6mHs/UcD3).

**PREPARATION OF MSC-CM**

The conditioned medium was performed by seeding MSCs between the passages 4 to 9, at a density of 3320 cells/cm^2^, on plastic culture flasks, to form a semi-confluence. After 24 h of culture with DMEM 10% FBS, the cells were washed once with PBS and covered with 120 µL/cm^2^ of serum-free DMEM medium to release factors for the next 24 h under an appropriate atmosphere (humidified, 5% CO_2,_ 37ºC). The resulting MSC-CM was collected and then filtered, through centrifugation (600 × *g*, 10 min) to eliminate the cellular debris. It was then stored at a temperature of -80°C until further use [(Iser et al. 2016)](https://paperpile.com/c/AA6mHs/Smnd).

**PROLIFERATION ASSAY**

Cell counting was performed by 0.4% trypan blue exclusion method. 1 × 10^5^ HaCaT cells was seeded in a 24-well plate, remaining for 24 h for cell adhesion. The monolayers were washed and exposed to the treatments: DMEM 10% FBS, MSC-CM, and serum-free DMEM remaining for 24, 48, 72 and 96 h. Cultures pretreated with 20 µg/mL mitomycin C (Sigma) for 4 h were also used as a complementary control for the two initial times. The last two groups had their medium changed on the second day, when the nonadherent cells were collected and counted. All cultures tested were washed with PBS, detached using 0.25% trypsin and 0.01% EDTA in PBS (*v/v*), and dissociated for counting. The numbers of viable and dead cells were assessed by counting with a Neubauer chamber under a phase-contrast microscope (DMi1, Leica, Wetzlar, German).

**SCRATCH WOUND ASSAY**

To proceed with the migration assay, HaCaT cells were plated on 24-well plates to obtain a final density of 1 × 10^5^ cells per well. The cells were then maintained at 37°C and under a humidified atmosphere of 5% CO_2_ for 24 h to allow cell adhesion and the formation of a confluent monolayer. The monolayers were then injured, along the diameter of each well, with a sterile pipette tip (100 µL) to leave a scratch of approximately 450 µm in width. The culture medium was then immediately removed, containing the dislodged cells, and each scratch was washed twice with PBS. The wells that presented homogeneous risks were selected and treated with 20 µg/mL mitomycin C in DMEM 10% FBS for 3 h under appropriate atmosphere for the cells. After a new wash, the cells were then covered by the treatments: DMEM, MSC-CM, DMEM 10% FBS, or by DMEM plus 10 ng/mL TGF-β1 (Sigma). The wound closure was monitored through phase-contrast microscopy (BX-50, Olympus), using a ×40 objective, at times 0, 24 and 48 h after lesion. The images were measured in four points, using the Image J software (NIH, Bethesda, MD, USA), after adjustments of the calibration blade. The measures were estimated in percentage, determining the initial time (0 h) as the 100% opening value of the lesion [(Iser et al. 2016)](https://paperpile.com/c/AA6mHs/Smnd).

**TRANSWELL MIGRATION ASSAY**

The migration of HaCaT cells was also evaluated during a co-culture with MSCs using transwell inserts (Greiner Bio-One, Kremsmünster, Austria). The amount of 2 × 10^4^ HaCaT cells were placed in the upper chamber of 8-µm pore size inserts, in a 24-well format. Cells were allowed to adhere for 4 h under a humidified atmosphere of 5% CO_2_. MSCs were seeded in the bottom chamber at 13.8 × 10^3^ cells/cm^2^, adhering for the same time. In the lower compartments, different chemoattractants were used: MSCs covered by DMEM medium; MSCs covered by 2 µM TGF-β1 receptor kinase inhibitor (SB 431542, Tocris Bioscience, Bristol, UK) in DMEM; 10 ng/mL of TGF-β1 factor in DMEM; or both together, 10 ng/mL TGF-β1 factor and 2 µM SB 431542 in DMEM. After a culture of 48 and 72 h, the inserts were removed, and the non-migratory part of the cells was cleaned by gently scrubbing a swab inside. The inserts were washed once with PBS (37ºC) and were fixed using ice-cold methanol for 15 min. After washing them with distilled H_2_O, staining was performed with 0.25% crystal violet in a solution of 20% methanol in H_2_O (*v/v*) for 10 min. The inserts were washed twice with H_2_O and allowed to dry completely. The membranes were removed and mounted in slides. Six captures were performed at ×200 magnification, under an optical microscope (Olympus), in order to count the migratory part of the HaCaT cultures [(Iser et al. 2016)](https://paperpile.com/c/AA6mHs/Smnd).

**ACTIN CYTOSKELETON STAINING**

The actin cytoskeleton reorganization was assessed via filamentous actin (F-actin) staining. Briefly, HaCaT cells were seeded on 13 mm glass coverslips to obtain a final density of 4 × 10^4^ cells per well, then were maintained for 24 h with the DMEM 10% FBS to allow cell adhesion. After this, the cells were treated for periods of 24 and 48 h, either with the MSC-CM or one of the controls: DMEM, DMEM 10% FBS, DMEM 10 ng/mL TGF-β1, and MSC-CM 2 µM SB 431542. After treatment time, the cells were washed once with PBS and were fixed with 4% paraformaldehyde in PBS (2-8ºC, 10 min). The cells then received 0.1% Triton-X100 (Sigma) in PBS for 30 min, working as a permeabilization buffer. The cytoskeleton marking was performed using a green-fluorescent Alexa Fluor 488 Phalloidin dye (Invitrogen, Carlsbad, CA, USA), for 40 min, at 2 to 8ºC, in a light-free environment. The images of the labeled actin fibers were captured using a fluorescence microscope (BX-50, Olympus) coupled with a 495-518 nm excitation/emission filters. To carry out the counting of filopodia protrusions at the apical side, the cultures were injured through scratch wound method, and after 24 and 48 h of migration, the cells were also stained and the number of visible filopodia sense the wound was counted [(Sous Naasani et al. 2019)](https://paperpile.com/c/AA6mHs/UcD3).

**NUCLEAR MORPHOMETRIC ANALYSIS (NMA)**

The nuclear morphology of the HaCaT cells was analyzed using the NMA software developed by Filippi-Chiela and co-authors (Filippi-Chiela et al., 2012). Briefly, after having undergone the processes of seeding and adhesion, cells were either treated with the DMEM 10% FBS, the serum free culture medium, or the MSC-CM for periods of 24 and 48 h, fixed and analyzed as already described [(Filippi-Chiela et al. 2012; Rodrigues et al. 2014; Iser et al. 2016)](https://paperpile.com/c/AA6mHs/0B3F+NlNe+Smnd).

**STATISTICAL ANALYSIS**

The data were analyzed through the one-way or two-way ANOVA followed by Bonferronis’s or Tukey’s multiple comparisons test, using the Graph Pad Prism 6 software (La Jolla, CA, USA). The results are expressed as mean value ± SEM (Standard Error). The differences were considered significant when the *p* value was of 0.05 or less.
